# Fungal–Bacterial Co-Infections and Super-Infections among Hospitalized COVID-19 Patients: A Systematic Review

**DOI:** 10.3390/jof9060598

**Published:** 2023-05-23

**Authors:** Farkad Bantun

**Affiliations:** Department of Microbiology, Faculty of Medicine, Umm Al-Qura University, Makkah 21955, Saudi Arabia; fmbantun@uqu.edu.sa; Tel.: +966-560345465

**Keywords:** co-infections, coronavirus, fungal–bacterial, severe acute respiratory syndrome, super-infections

## Abstract

This study systematically reviewed fungal–bacterial co-infections and super-infections among hospitalized COVID-19 patients. A PRISMA systematic search was conducted. On September 2022, Medline, PubMed, Google Scholar, PsychINFO, Wiley Online Library, NATURE, and CINAHL databases were searched for all relevant articles published in English. All articles that exclusively reported the presence of fungal–bacterial co-infections and super-infections among hospitalized COVID-19 patients were included. Seven databases produced 6937 articles as a result of the literature search. Twenty-four articles met the inclusion criteria and were included in the final analysis. The total number of samples across the studies was 10,834, with a total of 1243 (11.5%) patients admitted to the intensive care unit (ICU). Of these patients, 535 underwent mechanical ventilation (4.9%), 2386 (22.0%) were male, and 597 (5.5%) died. Furthermore, hospitalized COVID-19 patients have a somewhat high rate (23.5%) of fungal–bacterial co-infections and super-infections. Moreover, for SARS-CoV-2 patients who have a chest X-ray that suggests a bacterial infection, who require immediate ICU admission, or who have a seriously immunocompromised condition, empiric antibiotic therapy should be taken into consideration. Additionally, the prevalence of co-infections and super-infections among hospitalized COVID-19 patients may have an impact on diagnosis and treatment. It is crucial to check for fungal and bacterial co-infections and super-infections in COVID-19 patients.

## 1. Introduction

The respiratory illness COVID-19, which is the cause of the present COVID-19 pandemic, is brought on by the coronavirus known as SARS-CoV-2 [1]. On 30 January 2020, and 11 March 2020, respectively, the World Health Organization (WHO) labeled the outbreak a pandemic and a public health emergency of international concern [2]. As of 7 August 2022, 581.8 million confirmed cases of COVID-19 and 6.4 million deaths had been reported globally [3]. According to a meta-analysis, 17% of SARS-CoV-2 infections are asymptomatic, and asymptomatic individuals are 42% less likely to transmit the virus [4]. In addition, there is uncertainty about reinfection and long-term immunity [5].

Although reports have suggested that reinfection is happening with varying intensity, it is unknown how frequently it occurs [6]. The results of recent research have implications that immunizations may not be able to provide lifelong protection against the virus and that herd immunity may not be able to eradicate the virus if reinfection is a common occurrence [7].

On the other hand, it is uncertain how common co-infections and super-infections are among humans worldwide [8]. A co-infection is an illness that occurs at the same time as the first infection, but a super-infection is an infection that occurs after a previous infection, particularly those caused by microorganisms that are resistant to previously employed antibiotics [9,10]. Co-infections and super-infections with SARS-CoV-2 are usually caused by community-acquired bacteria, such as *Streptococcus pneumoniae, Hemophilus influenza*, or *S. Aureus,* or by hospital-acquired, multidrug-resistant bacteria and fungi [10]. Furthermore, bacterial and fungal co-infections and super-infections represent important complications of viral diseases and may be associated with worse outcomes [11]. In contrast to the well-documented phenomenon of co-infections and super-infections with bacterial, viral, and other pathogens in influenza, SARS, MERS, and other respiratory viral illnesses, information on bacterial and fungal co-infections and super-infections in SARS-CoV-2 patients is scarce and still developing [12].

Patients who have SARS-CoV-2 are at significant risk of contracting a nosocomial co-infection and may need to stay in the hospital for a long time, either in regular wards or the intensive care unit [13]. Additionally, SARS-CoV-2 patients may experience severe pneumonia, necessitating hospitalization, intubation, and transfer to the intensive care unit [14]. The likelihood of developing multidrug resistance increases when these co-infections are treated with empiric, broad-spectrum antibiotic therapy [13]. The global proportion of microorganisms that are resistant to multiple drugs has also been reported [15]. Additionally, the primary cause of death in this cohort is respiratory failure brought on by SARS-CoV-2 infection; nevertheless, multiple observations have shown that hospitalized SARS-CoV-2 patients may also be more vulnerable to co-infections and super-infections [16].

Additionally, co-infections and super-infections, particularly in countries with limited resources, are thought to play a role in the relatively high incidence of severe infection and mortality in SARS-CoV-2. These factors, along with a lack of natural immunity and viral replication in the lower respiratory tract, are thought to contribute to severe lung injury and acute respiratory distress syndrome [17]. Therefore, the aim of the current study was to systematically review fungal–bacterial co-infections and super-infections among hospitalized COVID-19 patients.

## 2. Methods

### 2.1. Protocol Registration

The protocol for this systematic review was submitted and registered with the International Prospective Register of Systematic Reviews (PROSPERO) (Code no. CRD42022368456). In addition, this systematic review followed the guiding principles of the Preferred Reporting Items for Systematic Reviews and Meta-Analyses (PRISMA) statement [18].

### 2.2. Eligibility Criteria

Articles that fulfilled the following eligibility criteria were included in this systematic review:

- Participants: patients of any age with a confirmed positive COVID-19 test who developed fungal–bacterial co-infections and super-infections during the hospital stay.
- Exposure: severe acute respiratory syndrome coronavirus 2.
- Outcome: fungal–bacterial co-infections and super-infections.

However, the exclusion criteria listed below were taken into consideration: (1) reviews; (2) non-English language articles; (3) case reports; (4) articles not reporting bacterial and fungal infection detection; (5) articles lacking critical information; (6) controlled case series; (7) editorials and commentaries; (8) articles without the full text.

### 2.3. Search Strategy

Systematic searches were conducted on the Medline, PubMed, Google Scholar, PsychINFO, Wiley Online Library, NATURE, and CINAHL databases between 1 January 2020 and 20 September 2022. The search was restricted to the English language restriction, without limitation on the region. A collection of search terms was produced using truncations, Medical Subject Headings (MESH), and Boolean operators (Table 1).

### 2.4. Data Extraction

The Mendeley reference management program was used to compile all articles from automated database searches. After deleting duplicates, screening was performed to ensure the studies met the qualification requirements. Articles were screened in three stages based on title, abstract, and full text. To facilitate the comparison and synthesis of studies, key information pertinent to the study’s focus was methodically gathered and collated. Table 2 lists the first author’s name, publication date, country of origin, the total number of SARS-CoV-2 positive patients who underwent co-pathogen testing, the total number of co-infected patients admitted to the intensive care unit (ICU), the total number of participants on mechanical ventilation, the total number of deaths, the total number of bacterial and fungal co-infections and super-infections, the types of organisms, and the total number of antimicrobials used. The study design, sample size, participant age, and key findings of each included article were extracted and are reported in Table S1.

### 2.5. Assessment of Quality

The National Institutes of Health Quality Assessment tool for Observational Cohort and Cross-Sectional Studies, which addresses the design, selection bias, data collection, confounders, blinding, and attrition, was used to evaluate the quality of the qualifying papers. Overall grades of ‘good’, ‘fair’, or ‘poor’ were provided for each article [41].

### 2.6. Data Analysis

We were unable to perform a meta-analysis since the outcome measures varied amongst the articles [42]. Rather, narrative synthesis was carried out. This made it possible to take into account confounding, mediating, and moderating variables, which are frequently neglected in meta-analyses. Each study was introduced before being compared, analyzed, and then synthesized.

## 3. Results

**Figure 1:**
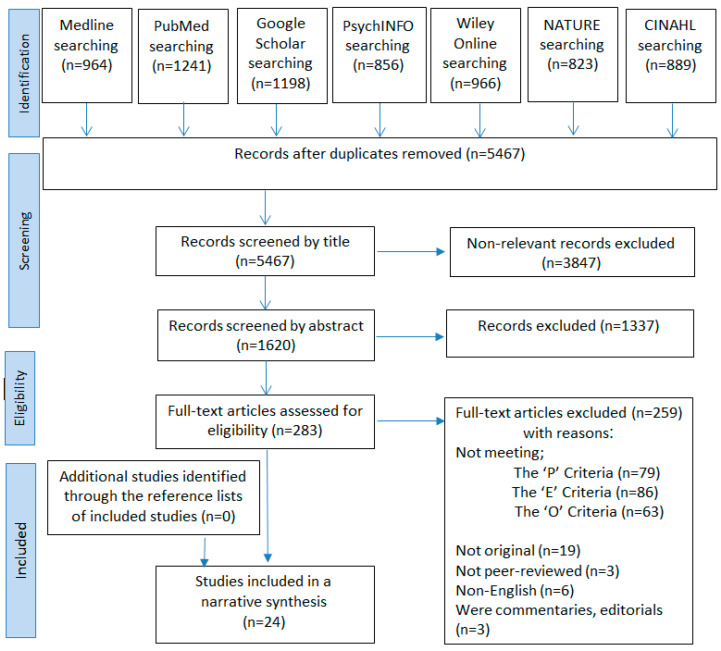
PRISMA flow diagram.

S databases used for the literature search produced 6937 articles. After 1470 duplicates were eliminated, 3847 unique articles were disqualified from the screening on the basis of the title. After screening the remaining 1620 articles in the abstract, 1337 were eliminated, leaving 283 articles. Two hundred fifty-nine articles were found to be ineligible after reviewing the entire contents, with not meeting the participant criteria being the most frequent cause. More information on the exclusion criteria is provided in the PRISMA flowchart [18] (Figure 1). In the end, 24 articles met the criteria for this systematic review.

P/E/O criteria: participants: exp patients/or admitted* OR hospitalized* OR infected* OR positive COVID-19; Exposure: Coronavirus infection* OR exp SARS coronavirus/or exp severe acute respiratory syndrome/OR COVID OR SARS; outcomes: exp mixed infection/OR ‘bacterial infection’ OR exp fungal infection/or exp co-infection/or exp co-infection/or co-infect/or exp super-infection/or exp super-infection* or exp coinfect/or exp concomitant infect/OR concurrent infection* OR exp mixed infect/or exp anxiety disorders/OR exp stress, psychological/or ‘psychological distress*.

### 3.1. Description of Articles

The reviewed articles are summarized in Table 2. Twelve articles were published in 2020, six articles were published in 2021, and six articles were published in 2022. In addition, six studies were conducted in China, four studies were conducted in the United Kingdom (U.K.), three studies were conducted in the United States (U.S.), three studies were conducted in Italy, two studies were conducted in Iran, and one study each was conducted in Pakistan, Egypt, Scotland, Saudi Arabia, Palestine, and Spain.

The participants’ ages ranged from 2 to 99. Three articles did not mention the age of the participants [6,21,31]. All articles included hospitalized patients. The total number of samples across the studies was 10,834, with a total of 1243 (11.5%) participants being admitted to the ICU; 535 underwent mechanical ventilator (4.9%), 2386 (22.0%) were male, and 597 (5.5%) died. All articles exclusively reported the presence of both bacterial and fungal co-infections and super-infections among SARS-CoV-2 patients. The antimicrobials used in the included articles were CEP (cephalosporin), CLR (clarithromycin), AZM (azithromycin), CAR (carbenicillin), TGC (tigecycline), LZD (linezolid), F.Q. (fluoroquinolones), β-Lactamase inhibitors, MET (methicillin), VAN (vancomycin), TZP (piperacillin-tazobactam), DOX (doxycycline), LVX (levofloxacin), CIP (ciprofloxacin), CRO (ceftriaxone), FEP (cefepime), and MEM (meropenem).

Laboratory techniques for co-pathogen detection within the articles included respiratory samples and RT-PCR tests; serologic tests (antibodies), RT-PCR tests with respiratory and/or blood cultures; respiratory and/or blood cultures; others tested both serology and RT-PCR, and others did not specify their testing methods. Additionally, data regarding the names of bacterial and fungal species were not mentioned in the included articles. Moreover, Wang et al. [19], Chen et al. [23], and Wang et al. [30] reported that 8, 15, and 5 antifungal agents were used among the study participants, respectively, but they did not specify the type of antifungal agents.

### 3.2. Quality Assessment

Nearly all of the articles had clearly defined objectives, but overall, the methodological quality was evaluated to be from poor to fair (Table 3). Four articles were scored as being of good quality [6,24,29,30], six articles were scored as being of fair quality [21,25,33,38,39,40], and the remaining fourteen articles were scored as being of poor quality [16,18,19,20,21,22,23,25,26,27,29,30,31,32,33,34,35,36,37].

All articles included retrospective cohorts in their design, except Ramadan et al.’s article, which used a prospective cohort [6]. The retrospective cohort design of 23 articles is susceptible to three common sources of bias: information, confusion, and interaction biases. In addition, a prospective cohort design could be affected by the loss of follow-up [43]. The majority of articles were single-center ones, and only four studies were multi-center ones.

## 4. Discussion

Co-infections and super-infections are typically caused by bacteria that have been acquired in the community or in a hospital. In addition, co-infections and super-infections may become more likely as a result of multidrug-resistant bacteria or fungi [10]. Furthermore, fungal–bacterial co-infections and super-infections are considered to be important complications of viral diseases and may be associated with worse outcomes [11]. This systematic review was conducted to examine the presence of fungal–bacterial co-infections and super-infections among hospitalized patients with SARS-CoV-2. In this review, all articles that exclusively reported the presence of fungal–bacterial co-infections and super-infections among hospitalized SARS-CoV-2 patients were included. Twenty-four articles met the inclusion criteria and were included in the final analysis. The included studies were conducted in China, the U.K., the U.S., Italy, Iran, Pakistan, Egypt, Scotland, Saudi Arabia, Palestine, and Spain. The total sample size of all the studies was 10,834.

### 4.1. Prevalence and Outcome

The main results of this review show that hospitalized SARS-CoV-2 patients have a somewhat high rate (23.5%) of fungal–bacterial co-infections and super-infections. However, the included articles often lack uniformity in both the reports and examinations of fungal–bacterial co-infections and super-infections, which may have under- or overestimated the rates of co-infections and super-infections. 

According to previous research, patients with severe viral infections frequently develop fungal infections caused by *Aspergillus*, *Candida*, *Cryptococcus neoformans*, *Pneumocystis*, or other fungal species, which is associated with apparent increases morbidity and mortality [40]. Additionally, due to severely damaged alveoli and a reduction in leukocyte counts, SARS-CoV-2 patients are susceptible to fungal infections at the later stages of the illness [43]. Comorbidities, immune-modulating therapies, widespread use of over-the-counter antibiotics, pathological aberrations of the immune system, and epithelial barriers caused by SARS-CoV-2 are likely to play a role as well [44]. A comprehensive review and meta-analysis study was undertaken by Chinese researchers on 2780 confirmed SARS-CoV-2 patients from nine relevant investigations. They found that Asian patients were more likely than patients in studies from the U.K. were to have a fungal co-infection, and from 0.12% to 0.15 percent of cases tested positive for a fungal infection following fungal culturing at admission [45]. Therefore, after the confirmation of SARS-CoV-2 infection, several researchers have proposed that patients should undergo routine screening for bacterial and fungal infections [46,47].

### 4.2. Antimicrobial Drugs

The findings of the current review show that the most commonly used antimicrobials in the included articles were CEP, CLR, AZM, CAR, TGC, LZD, F.Q., β-Lactamase inhibitors, MET, VAN, TZP, DOX, LVX, CIP, CRO, FEP, and MEM, which are highly recommended for use in patients with community bacterial pneumonitis needing admission to a hospital, and also in urinary tract infections, abdominal infections, and infections caused by other sensitive bacteria. In order to decrease the inappropriate use of empiric antibiotics, Hughes also investigated a method based on the stratification of individuals who are at risk of developing a bacterial co-infection or super-infection within the first 72 h of admission [48]. The outcomes of earlier research also showed the importance of beta-lactamase inhibitors in preventing the development of antibiotic resistance by blocking serine beta-lactamases, which are enzymes that deactivate the beta-lactam ring, which is a chemical compound shared by all beta-lactam antimicrobials [49]. However, the analysis of collected specimens from hospitalized SARS-CoV-2 patients revealed bacterial co-infection throughout the pandemic [37]. Therefore, empirical antibiotic therapy is a common component in SARS-CoV-2 treatment procedures to address potential organisms. When bacterial co-infections and super-infections are identified, especially if the patient is hospitalized in the intensive care unit, wide-spectrum antibiotic regimens are given [50]. There are currently no data available to compare SARS-CoV-2 patients who received antibacterial medicines with those who did not, which would allow us to assess the effectiveness of the treatment. Additionally, previous research has shown that repeated antibiotic doses have a minimal effect on the disease’s progression or the fatality rates of patients [48]. Additionally, the use of antibiotics is limited to cases of known or suspected bacterial infections. The current review also showed that empiric antibiotic therapy should be taken into account in SARS-CoV-2 patients who have a chest X-ray that indicates a bacterial infection and who need emergency ICU admission, or those who have a highly immunocompromised condition. Future studies are recommended, focusing on the specific usage of antimicrobial drugs, the differences between developing and non-developing countries, and the influences of other variables, including the effect of the socioeconomic variables on patients’ outcome.

### 4.3. Detection Techniques

RT-PCR tests with respiratory and/or blood cultures, serological tests (antibodies), serological and RT-PCR tests combined, and undisclosed testing methods were among the laboratory techniques for co-pathogen detection in the articles in the current review. Additionally, in the current systematic review, the majority (19 articles) used RT-PCR tests, only one article used both serology and RT-PCR tests, and the remaining four articles did not specify their testing methods. 

Incidences of co-infections and super-infections may have been underestimated or inflated as a result of the lack of standardization in laboratory techniques for detecting fungal–bacterial co-pathogens in the articles. Clinicians should maintain a high level of diligence for these co-infections and super-infections, especially in critically ill patients, given the current diagnostic challenges and uncertainties relating to the risks associated with fungal–bacterial co-infections and super-infections in hospitalized SARS-CoV-2 patients. Additionally, the French High Council for Public Health advised clinicians to focus on fungal infections in SARS-CoV-2 patients, especially in cases of a severe illness [51].

According to the results of the current study, 1243 (11.5%) of the patients with fungal–bacterial co-infections and super-infections were brought to the intensive care unit (ICU), of whom 535 (4.9%) required mechanical ventilation, and 597 (5.5%) passed away. Bardi et al. (2021) [33] investigated nosocomial infections associated with COVID-19 in the intensive care unit. He revealed that 91 confirmed nosocomial infections occurred in 57 patients while they were receiving ICU care. A large variety of Gram-positive (55%) and Gram-negative (30%) bacteria, as well as fungi (15%), were responsible for the majority of them, including pneumonia (23%), tracheobronchitis (10%), and urinary tract infections (8%). The next most frequent bloodstream infections were primary (31%) and catheter-related (25%) infections. The author also discovered that staying in the ICU for a significant amount of time increases the risk of co-infections and super-infections occurring for more than a week. In fact, patients who have co-infections and super-infections are more likely to need ICU care and mechanical ventilation [52]; the likelihood of developing co-infections and super-infections also rises as ICU stays lengthen. A secondary infection linked to a hospital setting is also more likely to occur when mechanical ventilation is required [53]. Additionally, super- and co-infections exacerbate patients’ prognoses and raise the fatality rate. As a result, doctors should be frequently sought out upon admission, and also, in each case where the radiologic and clinical statuses have worsened despite intervention with high-dose corticosteroids. This systematic review also found that the high rates of co-infections and super-infections were common among severely ill patients due to age and other predisposing factors, including COPD, asthma, diabetes, high cholesterol, and high blood pressure. More research is needed in the future to determine the underlying processes of fungal–bacterial co-infections and super-infections among hospitalized SARS-CoV-2 patients.

### 4.4. Strengths and Limitations

The main strengths of this review are that it is the first one to systematically examine fungal–bacterial co-infections and super-infections among hospitalized patients with SARS-CoV-2 and its large sample size. However, our study has a number of limitations. The included articles described often did not uniformly report or undertake examinations to detect fungal–bacterial co-infections and super-infections, which may resulted in under- or overestimated rates of co-infections and super-infections. In addition, the retrospective cohort design of the included articles reduced the control over multiple confounders and data collection, which could increase the potential for information, confusion, and interaction biases. Another drawback was that the current systematic review was conducted by a single author.

## 5. Conclusions

Hospitalized SARS-CoV-2 patients have a somewhat high rate (23.5%) of fungal–bacterial co-infections and super-infections. In addition, the prevalence of co-infections and super-infections among hospitalized SARS-CoV-2 patients may have an impact on diagnosis and treatment. Moreover, for future diagnostics and treatment options, it is crucial to check for fungal and bacterial co-infections and super-infections in SARS-CoV-2 patients using fungal and bacterial culture assays. Additionally, the use of antibiotics should be moderated and based on the findings of sensitivity and culture tests. Finally, it is advised to use infection control measures to avoid nosocomial infections.

## Figures and Tables

**Table 1:** Search terms and linkage (Medline).

Participants	exp patients/or admitted* OR hospitalized* OR infected* OR positive COVID-19
	AND
Exposure	Coronavirus infection* OR exp SARS coronavirus/or exp severe acute respiratory syndrome/OR COVID OR SARS
	AND
Outcomes	exp mixed infection/OR ‘bacterial infection’ OR exp fungal infection/or exp co-infection/or exp co-infection/or co-infect/or exp super-infection/or exp super-infection* or exp coinfect/or exp concomitant infect/OR concurrent infection* OR exp mixed infect/or exp anxiety disorders/OR exp stress, psychological/or ‘psychological distress*’

The asterisk (*) represents any group of characters, including no character.

**Table 2:** Summary of articles.

Study	Country	Patients with SARS-CoV-2 Who Underwent Co-Pathogen Testing: n	Patients with Co-Infection, n (%)	ICU Admissions, n (%)	Mechanical Ventilation, n (%)	Deaths, n (%)	Bacterial Co-Infection,n (%)	Fungal Co-Infection,n (%)	Organisms	Antimicrobials Use, n
Wang et al. (2020) [19]	China	57	9 (31.5)	NM	NM	5 (7.5)	5 (17.2)	2 (6.9)	Bacteria Fungi	39 F.Q. 8 Antifungal agents
Yang et al. (2020) [20]	China	53	7 (13.5)	52 (100.0)	37 (71.0)	32 (61.5)	4 (7.7)	3 (5.8)	Bacteria Fungi	NM
Zhu et al. (2020) [21]	China	257	243 (94.5)	3 (1.2)	0	0	236 (91.8)	60 (23.3)	Bacteria Fungi	NM
Falcone et al. (2020) [22]	Italy	315	69 (21.9)	56 (71.6)	43 (62.4)	13 (18.8)	11 (15.6)	5 (5.5)	Bacteria Fungi	20 DOX 9 AZM 27 CRO 23 TZP 2 F.Q.
Chen et al. (2020) [23]	China	99	5 (5)	23 (23)	17 (17)	11 (11)	1 (1)	4 (4)	Bacteria Fungi	7 CEP, CAR, TGC, and LZD 15 Antifungals
Hughes et al. (2020) [24]	UK	836	51 (6.1)	3 (5.9)	NM	NM	51 (6.1)	30 (3.6)	Bacteria Fungi	NM
Li et al. (2020) [25]	China	32	14 (43.7)	11 (78.6)	4 (28.6)	N.M.	10 (31.2)	7 (21.9)	Bacteria Fungi	NM
Intra et al. (2020) [26]	Egypt	260	28 (10.8)	60 (23.0)	8 (13.3)	24 (40.0)	28 (10.8)	5 (1.9)	Bacteria Fungi	28 CLR and AZM
Cataldo et al. (2020) [27]	UK	77	39 (50.6)	39 (100.0)	NM	NM	28 (36.4)	11 (14.3)	Bacteria Fungi	NM
Nasir et al. (2020) [28]	US	140	57 (40.7)	57 (100.0)	56 (98.0)	31 (54.0)	51 (36.4)	6 (4.3)	Bacteria Fungi	53 CEP 53 CLR and AZM 47 Other antibiotics
Ramadan et al. (2020) [6]	Italy	61	35	35 (100.0)	35 (100.0)	N.M.	13 (37.1)	19 (54.3)	Bacteria Fungi	17 β-Lactamase inhibitors, VAN, CAR, or MET
Sepulveda et al. (2020) [29]	Italy	NM	57	57 (100.0)	48 (84.0)	18 (32.0)	27 (47.4)	28 (49.0)	Bacteria Fungi	2 VAN 4 TZP 1 CAR
Wang et al. (2021) [30]	Pakistan	23	9 (39.1)	23 (100.0)	2 (22.2)	4 (17.4)	9 (39.1)	5 (21.7)	Bacteria Fungi	7 CLR and AZM 5 Antifungals
May et al. (2021) [31]	US	4185	159 (3.8)	NM	NM	NM	156 (3.7)	3 (0.07)	Bacteria Fungi	NM
Yang et al. (2021) [32]	UK	1396	37 (2.7)	11 (29.7)	N.M.	10 (27.0)	37 (2.7)	4 (0.3)	Bacteria Fungi	NM
Gerver et al. (2021) [33]	China	NM	20	20 (100.0)	12 (60.0)	N.M.	96 (100.0)	3 (42.9)	Bacteria	NM
Nori et al. (2021) [34]	UK	2279	879 (38.6)	NM	NM	202 (23.0)	404 (45.9)	475 (54.0)	Bacteria	NM
Bardi et al. (2021) [35]	US	4267	152	99 (65.0)	112 (74.0)	87 (57.0)	112 (73.7)	5 (3.6)	Bacteria Fungi	4130 DOX, AZM, LVX, CIP, CRO, FEP, VAN, and TZP
Shafiekhani et al. (2022) [36]	Iran	97	66 (68)	97 (100.0)	18 (18.6)	6 (6.2)	13 (13.4)	9 (9.3)	Bacteria Fungi	5 VAN 6 CRE
Ruiz-Rodriguez et al. (2022) [37]	Scotland	NM	30	30 (100.0)	25 (83.3)	10 (33.3)	9 (30.0)	10 (33.0)	Bacteria Fungi	NM
Alnimr et al. (2022) [38]	Saudi Arabia	1091	135 (12.4)	182 (17.9)	63 (92.6)	70 (6.4)	67 (6.6)	67 (6.6)	Bacteria Fungi	NM
Naseef et al. (2022) [39]	Palestine	458	321 (70.1)	321 (100.0)	N.M.	26 (8.1)	164 (51.1)	157 (48.9)	Bacteria Fungi	138 MEM and VAN 114 TZP and LVX
Nebreda-Mayoral et al. (2022) [40]	Spain	712	113 (15.9)	50 (7.0)	50 (44.0)	43 (38.0)	39 (5.0)	80 (11.0)	Bacteria	26 TZP 21 CAR 20 LZD 15 LVX
Shafiekhani et al. (2022) [36]	Iran	66	14 (21.2)	14 (100.0)	5 (35.7)	5 (35.7)	8 (57.1)	14 (100.0)	Bacteria Fungi	VAN MET CAR

NM: Not mentioned. The antimicrobials used in the included articles were CEP (cephalosporin), CLR (clarithromycin), AZM (azithromycin), CAR (carbenicillin), TGC (tigecycline), LZD (linezolid), F.Q. (fluoroquinolones), β-Lactamase inhibitors, MET (methicillin), VAN (vancomycin), TZP (piperacillin-tazobactam), DOX (doxycycline), LVX (levofloxacin), CIP (ciprofloxacin), CRO (ceftriaxone), FEP (cefepime), and MEM (meropenem).

**Table 3:** Quality assessment.

Study	Quality Rating	Quality Appraisal Findings
Wang et al. (2020) [19]	Poor	Retrospective cohort Single-center study Small sample size and not justified (n = 57)
Yang et al. (2020) [20]	Poor	Retrospective cohort Single-center study Small sample size and not justified (n = 52)
Zhu et al. (2020) [21]	Fair	Retrospective cohort Single-center study Sample size was satisfactory and justified (n = 257)
Falcone et al. (2020) [22]	Poor	Retrospective cohort Single-center study Small sample size and not justified (n = 69)
Chen et al. (2020) [23]	Poor	Retrospective cohort Single-center study Small sample size and not justified (n = 99)
Hughes et al. (2020) [24]	Good	Retrospective cohort Multi-center study Sample size was satisfactory and justified (n = 836)
Li et al. (2020) [25]	Fair	Retrospective cohort Multi-center study Small sample size and not justified (n = 32)
Intra et al. (2020) [26]	Poor	Retrospective cohort Single-center study Small sample size and not justified (n = 35)
Cataldo et al. (2020) [27]	Poor	Retrospective cohort Single-center study Small sample size and not justified (n = 57)
Nasir et al. (2020) [28]	Poor	Retrospective cohort Single-center study Small sample size and not justified (n = 23)
Ramadan et al. (2020) [6]	Good	Prospective cohort Multi-center study Sample size was satisfactory and justified (n = 260)
Sepulveda et al. (2020) [29]	Good	Retrospective cohort Multi-center study Sample size was satisfactory and justified (n = 4185)
Wang et al. (2021) [30]	Good	Retrospective cohort Multi-center study Sample size was satisfactory and justified (n = 1396)
May et al. (2021) [31]	Poor	Retrospective cohort Single-center study Small sample size and not justified (n = 77)
Yang et al. (2021) [32]	Poor	Retrospective cohort Single-center study Small sample size and not justified (n = 77)
Gerver et al. (2021) [33]	Fair	Retrospective cohort Single-center study Sample size was satisfactory and justified (n = 879)
Nori et al. (2021) [34]	Poor	Retrospective cohort Single-center study Small sample size and not justified (n = 152)
Bardi et al. (2021) [35]	Poor	Retrospective cohort Single-center study Small sample size and not justified (n = 140)
Shafiekhani et al. (2022) [36]	Poor	Retrospective cohort Single-center study Small sample size and not justified (n = 97)
Ruiz-Rodriguez et al. (2022) [37]	Poor	Retrospective cohort Single-center study Small sample size and not justified (n = 30)
Alnimr et al. (2022) [38]	Fair	Retrospective cohort Single-center study Sample size was satisfactory and justified (n = 1091)
Naseef et al. (2022) [39]	Fair	Retrospective cohort Single-center study Sample size was satisfactory and justified (n = 321)
Nebreda-Mayoral et al. (2022) [40]	Fair	Retrospective cohort Single-center study Sample size was satisfactory and justified (n = 712)
Shafiekhani et al. (2022) [36]	Poor	Retrospective cohort Single-center study Small sample size and not justified (n = 30)

## Data Availability

Not applicable.

## Supplementary Materials

The following are available online at https://www.mdpi.com/article/10.3390/jof9060598/s1, Table S1: The key findings of the included article.
