# Supplementary material for: Fungal–Bacterial Co-Infections and Super-Infections among Hospitalized COVID-19 Patients: A Systematic Review
Source: J Fungi (Basel). 2023 May 23;9(6):598. doi: 10.3390/jof9060598 (PMC10299597; doi:10.3390/jof9060598)
Supplement: Supplementary file 1 [file jof-09-00598-s001.zip › jof-2246107-supplementary.pdf]

Supplemental data

Table S1: The key findings of the included article

Study	Design	Age (Years)	Male, n (%)	Findings
Wang et al. (2020) [19]	Retrospective case series, single center	The majority (51%) were in the range (49–30)	NM	Sputum and blood were the sources of the patient samples evaluated for co-pathogens.
Yang et al. (2020) [20]	Retrospective case series, single center	The majority (73%) were in the range (79–50)	NM	Hospital-acquired illnesses were brought on by the identified germs.
Zhu et al. (2020) [21]	Retrospective cohort, single center	The median (IQR), 51 (99−2)	(53.7) 138	Patients aged 15–44 and under 15 had the highest and lowest incidence of co-infections, respectively. The majority of co-infections happened 1-4 days after the COVID-19 disease started. In cases of severe COVID-19, the percentage of viral, fungal, and bacterial co-infections was highest.
Falcone et al. (2020) [22]	Retrospective cohort, single center	The median age was 71 years (IQR 60−78.5)	51 (73.9)	Infections caused by several microbes made up 18.3%. The following factors were found to be significant predictors of superinfections: intestinal colonization by CAR-resistant Enterobacterales (OR 16.03, 95% CI 6.5-39.5, $P < 0.001$), invasive mechanical ventilation (OR 5.6, 95% CI 2.4-13.1, $P < 0.001$).
Chen et al. (2020) [23]	Retrospective case series, single center	Tme mean (SD), 55.5 (13.1)	67 (67.7)	Six individuals (6%) had elevated procalcitonin levels.
Hughes et al. (2020) [24]	Retrospective case series, multi-center	The median (IQR), 69 (55–81)	519 (62.0)	In the initial stages of hospital admission, the rate of bacterial co-infection in SARS-CoV-2 patients was low.
Li et al. (2020) [25]	Retrospective case series, multi- center	The median (IQR), 57 (47–69)	11 (78.6)	Patients with co-infections of SARS-CoV-2 were admitted to the ICU more frequently ($p < 0.05$), displayed more severe respiratory problems, and were more likely to develop sequelae such ARDS and shock.

Intra et al. (2020) [26]	Prospective cohort, multi-center	The most common age range was between 51and 70 years (%36.2)	144 (55.4)	Co-infections were found in 20 (71.4%) severe COVID-19 patients and eight (28.6%) moderate cases of co-infection.<br>Co-infections contributed to 25% of SARS-CoV-2 patients' deaths, and co-infected patients' SARS-CoV-2 complications and severity were both more severe.<br>In Upper Egypt, bacterial co-infection and antibiotic resistance are frequent among COVID-19 patients.
Cataldo et al. (2020) [27]	Retrospective cohort, single center	NM	NM	There was no association between hospital mortality with early or any respiratory sample pathogen isolation (p = 0.512 and p = 1.0, respectively).
Nasir et al. (2020) [28]	Retrospective case series, single center	The median (IQR), 63 (60–68)	47 (82.0)	The APACHE II score was substantially correlated with co-infection, which occurred a median of 9 days (IQR 5-11) after admission (p = 0.02).<br>Co-infection was substantially linked to both a longer ICU stay and death (OR 2.7, 95% CI 1.2-5.9, p = 0.015).
Ramadan et al. (2020) [6]	Retrospective cohort, single center	NM	NM	Severe COVID-19 patients had a higher prevalence of fungal and P. aeruginosa colonization than non-COVID-19 cases, it was found.
Sepulveda et al. (2020) [29]	Retrospective cohort, single center	The mean age (± standard deviation, SD) was 62 ± 13) years	41(72.0)	The following are probable major contributors to the high occurrence of bacterial and fungal infections in COVID-19 patients: 1) The significant COVID-19 immunological dysregulation; 2) the widespread use of antibiotics; and 3) the lack of commitment to infection control and prevention (IPC) methods.
Wang et al. (2021) [30]	Retrospective case series, single center	The median (IQR), 71 (51–85)	7 (77.8)	Patients with moderate to severe COVID-19 experience the complication of invasive aspergillosis.
May et al. (2021) [31]	Retrospective cohort, multi-center	NM	NM	When compared to COVID-19-negative patients, the rate of bacteremia was considerably lower in COVID-19 patients (3.8% vs. 8.0%; p 0.001). Within four days of incubation, more than 98% of all positive cultures had been discovered.<br>True bacteremia was most frequently caused by E. coli (16.7%), S. aureus (13.3%), K. pneumoniae (10.0%), and Enterobacter cloacae complex (8.3%) in COVID-19 patients.
Yang et al. (2021) [32]	Retrospective cohort, multi-center	The median (IQR), 76 (82–64)	28 (75.7)	When compared to SARS-CoV-2 patients without co-infections, ICU admission and mortality did not differ between the groups [215 (15.8%) vs. 11 (29.7%), p = 0.075] and [410 (30.2%) vs. 10 (27.0%), p = 0.68], respectively.<br>Within 48 hours of admission, bacterial co-infection was uncommon in COVID-19 patients who were hospitalized.
Gerver et al. (2021) [33]	Retrospective cohort, single center	The median age of 39 years (interquartile range, IQR, 36e67 years).	9(45.0)	More P. aeruginosa was found in the initial stages of ICU admission. Staphylococcus aureus and Acinetobacter baumannii were more frequently found with late ICU admission. Compared to the severe group, the critical group's fungal serum antigens

		the critical group with a median age of 69 years (IQR, 64e80 years)		were more commonly positive, and the frequency of positive fungal serum antigens rose with length of ICU stay.
Nori et al. (2021) [34]	Retrospective cohort, single center	The median 70 years [IQR:58-81]	524(59·6)	Escherichia coli, Staphylococcus aureus, and Klebsiella pneumoniae were the most typical pathogens. A higher number of cases with secondary infection were of Black or Asian ethnicity than cases without (67% vs. 41% and 99% vs. 82%, respectively, p0001). Cases with co/secondary infections were also older than cases without (median 70 years [IQR:58-81] vs. 55 years [IQR:38-77]).
Bardi et al. (2021) [35]	Retrospective cohort, single center	The median age of 62 years (IQR 52.5-72)	90(59.0)	Only 5% of patients had bacterial and fungal co-infections, but because they often affect the most vulnerable individuals, they are a serious issue.
Shafiekhani et al. (2022) [36]	Retrospective cohort, single center	The mean±SD age of the patients was 55.22±9.91 years	39(59.1)	During their COVID-19 course, 21% of the patients experienced co-infection at least once. Co-infections with fungi and bacteria led to much greater mortality. The most typical locations for pathogen isolation were urine and sputum (45.45% and 36.36%, respectively). VAN-resistant Enterococci (30%) were the primary cause of infections. With 23.3%, Escherichia coli was in second place.
Ruiz-Rodriguez et al. (2022) [37]	Retrospective cohort, single center	The mean±SD age of the patients was 58±11 years	24 (80.0)	A direct correlation between the abundance of a certain microbial species and microbial expansion (high biomass). Pathogen proliferation correlated with the patient's antibiotic treatment's selective pressure.
Alnimr et al. (2022) [38]	Retrospective cohort, single center	The median age for all patients were 48 years±4.3.	54 (79.4)	Gram-negative pathogens (Enterobacterales, Pseudomonas aeruginosa, and Acinetobacter baumanni) made up more than 50% of the etiological agents, and co-infection was observed in 67/68 non-survivors.
Naseef et al. (2022) [39]	Retrospective cohort, single center	170 (48.9%) were41-64 years of age	(50.5) 162	51.1% of isolates were co-infected with bacteria, mostly gram-negative isolates (Enterobacter species and K.pneumoniae). A. fumigatus caused a 48.9% fungal co-infection rate and an 8.1% death rate.
Nebreda-Mayoral et al. (2022) [40]	Retrospective cohort, single center	Their median age was 73 years (IQR 57−89)	420(59.0)	An important contributing reason to the rises in infection rates and morbidity among ICU patients was the A. baumannii outbreak.
Shafiekhani et al. (2022) [36]	Retrospective cohort, single center	The mean±SD age of the patients was 55.22±9.91 years	11(78.5)	Co-infections with fungi and bacteria led to much greater mortality. The most typical locations for pathogen isolation were urine and sputum (45.45% and 36.36%, respectively).
